# Voltaic Electricity and Amalgam Fillings

**Published:** 1872-09

**Authors:** E. C. Chase


### ARTICLE VI.

### *Voltaic Electricity and Amalgam Fillings.*

BY E. C. CHASE, D. D. S.

Every reader of this Journal knows that the only requisites of a voltaic battery are two dissimilar metals, connected by a conductor, immersed in acidulated water.

The common experiment of placing a piece of zinc and a copper coin in the mouth and then bringing them in contact, affords an illustration of a battery of this kind. While the metals are separated from each other nothing unusual is observed, but as soon as they are brought in contact a decided metalic taste is experienced, showing the generation of electricity..

Place a strip of zinc covered with mercury in a glass vessel, and opposite to it but not in contact with it, put a sheet of copper, and then fill the vessel with water and sulphuric acid, six or eight parts of the former to one of the latter. Now in connecting these two metals at the top with a copper wire, we will at once have a current of electricity established, flowing from the zinc through the liquid to the copper, and over the wire to the zinc again.

Hydrogen will be liberated at the copper plate in considerable quantities, while the connection between the two metals is maintained by the copper wire, or while the battery is in operation. The zinc is amalgamated to prevent the acid from acting upon it, and the impurities, such as particles of iron or other metals that it might contain, and thus forming numberless little batteries all over the surface of the zinc.

Now by the indiscriminate use of amalgam for filling teeth, we as dentists construct more voltaic batteries right in the mouths of human beings than we would care to acknowledge, and if they were but as powerful as they are numerous we would be enabled easily to distinguish an amalgam patient from a gold one, by the incessant flashes and innumerable sparks of electrical light, followed by constant peels and rumbling of thunder which could be seen and heard, proceeding from the mouth on any dark night, but happily for us, yet to the sorrow of the patient, the electric force confines itself to the teeth and contents of the mouth, instead of making the latter a luminary to guide the poor credulous victim of misplaced confidence to a land "flowing with milk and honey," as we are told did the pillar of fire in ancient days.

I say the indiscriminate use of amaglam. There are times and places when we are not only justified but compelled to use it from the nature of the case, and to neglect to do so would be morally criminal; again, there are other times when it would be equally criminal to make use of the stuff. Here is a case. We have, if you please, the distal surface of the first right superior molar to fill; the inside surface of the second molar of the same side and jaw, is already filled with gold. We have our cavity prepared, and the patient places full confidence in our ability and honesty as a dentist and a gentleman, and expects us to use our judgment in regard to the material for filling; expects and is willing to pay for the *best.* Now what shall we do? We know that to put in a good gold plug will tax us to the utmost of our skill; will require perhaps an hour or more of hard labor, both mental and physical. We are in a hurry, having a country practice, two or three are waiting "to have some teeth filled as they have eight or ten miles to go to-night," and it is now the middle of the afternoon. We know that we can fill the cavity with amalgam in two minutes with very little inconvenience either to ourself or the patient. We choose the latter course, consoling our conscience, if we have one, that just as likely as not the gold filling would have got wet if we had attempted it.

The filling is completed and the patient dismissed. Perhaps we never imagine that we have set up in that credulous mouth an apparatus which will destroy one or both of those teeth, We do not know that those elements of mercury, and silver, and tin, on the one hand; and the gold on the other, arranged in the manner we have them, and constantly moistened with acid saliva, constitute a machine capable of generating a fluid that not only has the power of overcoming the chemical affinities existing between the elements composing the tooth substance, but by its subtle action cause harmless bodies to so join in chemical union as to form the most powerful and destructive agents. This battery is in operation, the chloride of sodium ever present in the mouth is decomposed, the chlorine set free. Sulphuretted hydrogen and ammonia, probably, are present, resulting from the putrefactive decomposition of some organic matter left in the mouth, they are decomposed and abundance of hydrogen is liberated. Now these two elements, chlorine and hydrogen, bearing an ever burning love for each other, and affinities, that can never die, do not long remain in "single blessedness," but right now and here vow eternal fidelity, and are joined in matrimonial union, and now under a new name, *hydrochloric acid*, they fight side by side, becoming a powerful foe to the integrity of the tooth. Thus again is verified the old saying that "in union there is strength." Our patient returns in six months or a year, and we find to our chagrin the best proof of the ravages of some subtle force; both the gold and the amalgam plugs are badly decayed around the margins and their removal is necessary.

We may plead ignorant of the cause of this, but ignorance of a scientific truth in this day is a poor defence and will not stand. But suppose we have been taught the danger of such operations and did not heed the council, who then, indeed can measure our guilt?—*Missouri Dental Journal.*

